# Why Is Caries Prevention in Children So Difficult? A Narrative Opinion

**DOI:** 10.3390/ijerph21101316

**Published:** 2024-10-03

**Authors:** Svante Twetman

**Affiliations:** Department of Odontology, Faculty of Health and Medical Sciences, University of Copenhagen, Nørre Allé 20, 2200 Copenhagen, Denmark; stwe@sund.ku.dk

**Keywords:** access to care, caries prevention, children, fluoride, motivational interviewing, primary care, risk assessment

## Abstract

Caries is among the most common non-communicable diseases worldwide, yet it is commonly described as preventable. Caries prevention is, however, difficult and complex, since the disease has strong social, parental, behavioral, political, medical/genetic, and psychological elements, and the payment models are targeted at traditional conservative care. The aim of this paper is to discuss some key issues that make caries prevention in children be perceived as “difficult”: i) the communication gap between researchers and clinicians, creating unrealistic expectations of intervention efficacy; ii) the skewed distribution of caries and the problem of reaching children with the highest need; iii) limited access to care, which is a threat to oral health, in particular in low-socioeconomic-status, underserviced, and remote communities; and iv) the need to adopt behavior change models to affect the modifiable risk factors that are shared with other non-communicable diseases. Dentists cannot simply rely on fluoride exposure; proper education and training in caries risk assessment, behavior change models targeted at oral hygiene and sugar intake, and collaboration with primary healthcare and local school authorities are avenues that aid in caries prevention and reduce the uneven burden of the disease. Online education and mobile apps may help to promote oral health in areas with shortages of dental work force.

## 1. Introduction

Caries is a common non-communicable disease, affecting at least 500 million children worldwide [1]. As an average, early childhood caries affects 48% of all preschool children, ranging from 30% in Africa to 82% in Oceania [2]. The early stages of the disease are symptomless, but advanced or untreated caries is linked to a reduced quality of life in terms of pain, infections, eating problems, learning difficulties, and malocclusions [3]. The risk factors are well established, and it is commonly stated that “caries largely is a preventable disease” in textbooks and scientific papers [1,4]. The pertinent question is, therefore, why is caries prevention so difficult? One answer is that caries in childhood is a social, parental, behavioral, political, medical/genetic, psychological, economic, and dental problem. It is of course naive to think that dentists and other dental professionals can master all those skills in “one person”. Traditionally, dentists are trained to surgically treat caries and its sequelae with fillings, root canal treatment, and extractions, and they are less educated in behavior change theories and public dental health measures [5]. Moreover, factors like genetics, politics, reimbursement systems, and socioeconomic inequalities are not modifiable for a busy clinician. The benefits of a preventive approach are, however, not only less caries and improved child health and wellbeing but also reduced financial burdens on families and healthcare systems [6]. In this context, dental professionals have a shared responsibility and a key role to play. The aim of this article is to discuss some of the factors that make caries prevention in children “difficult”, or at least less successful, and point out realistic areas of improvement.

### 1.1. Communication Gap and Unrealistic Expectations?

**Figure 1:**
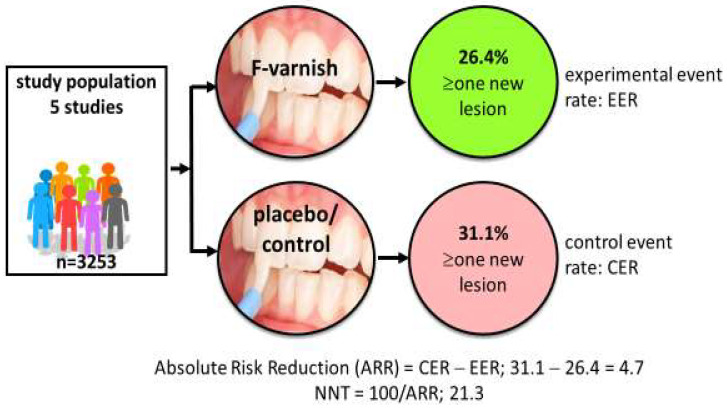
Calculation of number needed to treat (NNT). Data based on five randomized trials involving 3253 children indicate that 21 children must be treated with professional fluoride varnish applications 2–4 times per year for two years in order to prevent new caries development in one child. Data extracted from Marinho et al. [10].

Systematic reviews and consensus reports have displayed high- and moderate-certainty evidence for a palette of caries preventive measures in children, claimed to be “effective” [7,8]. Examples of such methods are tooth brushing twice daily with fluoride toothpaste and professional applications of fluoride varnish for those with increased caries risk [9,10]. But do researchers and clinicians understand “*effective*” in the same way? Researchers tend to look for effect sizes that are statistically significant (effective) while clinicians look for clinical significance (does it help my patients?). The gap between a test and placebo/control intervention is commonly expressed as the mean difference in decayed, missing, and filled primary (dmft) or permanent teeth (DMFT), or the standardized mean difference. A typical example is that preschool children treated with fluoride varnish every sixth month over two years on average have 37% fewer new decayed tooth surfaces than the placebo group [10]. This is indeed statistically significant on a group level but may be less impressive for clinicians who see their patients as individuals and consider whether or not the intervention actually prevents or arrests the disease. In some studies, individual data are available in terms of the proportion of children with new caries lesions in the test (experimental event rate) and the control group (control event rate), respectively. This allows for a calculation of the number needed to treat (NNT); an example is provided in Figure 1. NNT is the average number of patients you need to treat in order to avoid one additional bad outcome. The value allows for a straightforward communication of the science that gives clinicians insights on what it takes to earn “one caries-free child” in terms of time, resources, and spending in their local context. Notably, NNT is dependent on the level of caries in the population; 1.6 children need to brush with fluoride toothpaste (rather than a non-fluoride toothpaste) over three years to prevent one decayed, missed, or filled tooth surface in the young permanent dentition (DMFS) in populations with a mean caries increment of 2.6 DMFS per year [9]. In populations with a lower caries increment (1.1 DMFS per year), 3.7 children must use a fluoride toothpaste for three years to avoid one DMFS. The good message is, therefore, that caries-preventive measures in general tend to be more cost-effective in high-caries populations. It is, however, important to point out that NNT is context-dependent and should not be interpreted as a guarantee of treatment success for individual patients. A realistic understanding of treatment outcome is important so clinicians do not refrain from, or even abandon, evidence-based preventive measures.

To overcome the communication gap between scientists and clinicians, it would be helpful if clinical caries trials reported a consensus-agreed core outcome set [11] and that the results were also “translated” into a clinical relevance section. An example of such a development of a core outcome set in orthodontic research has been described by Tsichlaki and co-workers [12]. Randomized clinical trials should also be designed with health-economic aspects from the start in order to make local and long-term cost–benefit analyses of the intervention under study. Finally, an embedded qualitative research approach would be of interest in order to unveil the perceived value of the preventive measures to the patients, parents, and clinicians.

### 1.2. Skewed Caries Distribution

Despite the recognition of oral health as a human right, children throughout the world face inequalities in oral health care. The prevalence and experience of caries varies largely across countries, regions, and cities and between populations of different age groups, gender proportions, cultural backgrounds, socioeconomic status, and education level [1]. A skewed distribution is commonly observed, indicating that 20% of the population has 80% of the disease [13]. The principal avenues to successfully prevent a disease that is unevenly distributed are traditionally polarized as the “population strategy” versus the “high-risk strategy” [14,15]. The former targets all children in a community while the latter focuses on those with the highest need. There are numerous successful examples of both strategies; in Australia, for example, water fluoridation has reduced dental caries by 26–44% in children, regardless of age, income, or access to dental care [16]. On the other hand, fluoride varnish application together with oral health education was shown to be an effective approach for preventing caries in children living in economically disadvantaged rural areas in China [17]. The high-risk approach may look most reasonable in an era of declining caries, but it has also been criticized, since he majority of all new lesions actually occur in children classified as low-caries-risk [18]. There are also examples of unintended effects following the population-based strategy, as a school-based oral health education program may increase caries inequalities [19]. Thus, there is obviously no “one way fits all” and the best combination of the population and risk-based strategies must therefore be locally established depending on social and professional resources and health-economic systems. In this context, national initiatives, such as the Childsmile program in Scotland, have been internationally recognized. The program is incorporated into the national dental service contract and child health surveillance for all children up to 12 years of age. It employs an overarching population-based program of nursery and school-based tooth brushing, oral health improvement initiatives, and clinical prevention. Importantly, more intensive support is provided for children at high risk of caries. Although certain health inequalities remain after 10 years, the implementation of Childsmile has been associated with major improvements in child oral health [20].

A barrier is that most dentists seem reluctant to conduct caries risk assessment in children as a routine procedure and to benefit from validated risk assessment tools [21,22], most likely due to suboptimal education and clinical training [23]. The sparse use of risk assessment may be understood in the light of the relatively modest accuracy and performance of most models, but they are still a recommended procedure due to enhanced objectivity, consistency, and documentation [24]. One example that enables dentists to add structure to their clinical work is the CariesCare practice guide [25]. CariesCare was designed to help practitioners to deliver optimal caries care for their patients and is based on a four-step process; (i) Determine caries risk; (ii) Detect lesions, stage the severity, and assess their activity status; (iii) Decide on the most appropriate care plan for the specific patient at that time; and (iv) Do the preventive and tooth-preserving care needed. This “4D cycle” has the potential to both prevent and control caries, and dentists can engage parents and children in becoming long-term health partners in their practice [25].

The most important concern is, however, the lack of firm evidence on how to prevent caries among the most difficult and caries-active ten percent of the child population. This remains a knowledge gap, but a call for more and better research is problematic. Children with the highest risk, as well as their parents, are very difficult to enroll and retain for years in clinical trials, and it is also hard to ensure and maintain compliance. This has been denoted as the “inverse care law”, meaning that children with the highest need tend to be least likely to show up and utilize the preventive care that is offered [26]. Furthermore, since fluoride is regarded as an essential dental medicine [27] and “best clinical practice”, ethical committees around the world request at least twice daily tooth brushing as minimum “treatment as usual” in any control group. This narrows the gap to other active interventions and will require larger study populations, extended durations, and, in most cases, also external financing.

### 1.3. Access to Care

In an ideal word, all children would have unlimited access to dental care and dentists would provide high-quality treatment at low costs. In the real world, limited access to care is a threat to oral health, in particular in low-socioeconomic-status, underserviced, and remote communities [28]. Children living in such communities are less likely to visit oral health care providers, even if available, and they often lack knowledge and healthy behavior. For example, preschool children living in rural areas and rated as high-risk individuals are more likely to develop new caries than their urban peers [29]. While physical barriers, such as lack of adaption for children in wheelchairs and facilities for children with special needs, are mostly manageable, geopolitical factors and systems for provider remuneration may hamper access to care. Fee-for-service payments have been criticized for not promoting preventive care, while capitation payment plans may tend to support prevention but generate less frequent visits, later restorations, and opting out of difficult and non-cooperative child patients [30,31,32]. Thus, both payment systems have shortcomings in terms over- and under-treatment but dental professionals still have a responsibility to deliver appropriate and high-quality preventive and restorative care, irrespective of the remuneration system.

In many countries worldwide, there is an uneven distribution of dental professionals. In large cities, dentists are over-established, while there is a shortage of dental workforce in rural and remote areas. There are no firm evidence that access to care per se is linked to a higher prevalence of caries [33]; in fact, a systematic review has indicated that Africa has a lower prevalence of early childhood caries than the global pooled number, despite a generally low access to dental care [2]. The relationship between caries and access to care is, however, commonly confounded by socioeconomic and educational levels, as caries disproportionally affects poorer and marginalized groups in society [1]. A common explanation is the higher intake of free sugars and a lower use of fluorides in vulnerable families [5]. In addition, different incentive structures have implications on how patients are treated regarding state-of-the-art dental care. In urban areas, where dentists are over-established, dentists compensate for the fall in demand and their loss of income by raising their fees, recalling their patients more often, and “over-treating” caries in terms of restorative care [34]. This means that caries prevention is not likely on the agenda or has a low priority. In rural areas with a shortage of dental personnel, patients are put on waiting lists, only emergencies are handled, and no follow-ups are offered. Also in this scenario, caries prevention is likely to be downgraded for pragmatic reasons. A national risk-based recall system in which children with higher need receive dental recalls systematically more frequently than children with lower need may, however, substantially increase the proportion of treatment sessions including preventive care [35]. This was illustrated by a project in Sweden; socioeconomic inequalities in dental caries were reduced following the introduction of a regional risk-based capitation model for children in which more money was allocated to dentists working in deprived communities [36]. Evidence seems to indicate that further education and training of dentists coupled with a fair pay scheme would be a reasonable approach to favor the provision of caries-preventive measures [37]. Thus, enabling access to dental care can arguably improve health outcomes, reduce healthcare utilization costs, and reduce caries inequalities among children [38].

A limited access to dental care calls for collaboration with other health professionals and school authorities. Using the school as an arena for oral health promotion is certainly one way to overcome the shortage of dental work force. Examples are school-based supervised tooth brushing programs, fluoride mouth rinses, and fluoride varnish applications in low-socioeconomic-status areas with a large proportion of children with elevated caries risk. Such activities must, however, always be anchored with both custodians and teachers, and for fluoride deniers and parents worried about the impact of fluoride on neurological development in children, dental professionals must be prepared to provide fluoride-free alternatives. Properly trained teachers can also help with parental engagement, and there is currently low-certainty evidence that school-based interventions can be cost-effective among primary school children in low- and middle-income countries [39,40]. Concerning the inter-professional skill mix, there are multiple examples that caries prevention incorporated into nursing practice can reduce oral health disparities in children, especially among those living in poor or disadvantaged communities. In a systematic review covering eighteen programs for young children integrated into nursing and midwifery practice, all demonstrated positive oral health outcomes, including a reduction in caries [41]. Thus, the integration of oral health and caries prevention in primary healthcare is promising, but there are several obstacles to overcome. Health professionals frequently point on organizational barriers, lack of time, poor resources, inadequate funding, and/or insufficient oral health training [42]. The latter is important since parents’ oral health literacy must be specifically addressed. Also, parents may have concerns and question the unclear role of general health professionals when it comes to oral health [42]. In any case, the integration of oral health with the primary healthcare and school authorities is a beneficial approach that can be improved in many societies but requires further practice-based research in order to unveil the benefits and harm associated with health promotion and primary caries prevention conducted by non-dental personnel.

Another interesting novel avenue to compensate for a shortage of dental workforce in rural populations is to utilize online oral health education programs, delivered in a culturally and linguistically sensitive manner [43]. In addition, mobile apps may be useful in improving the oral health knowledge of parents/caregivers, aiding them in incorporating good oral habits into their children’s daily routines [44].

### 1.4. Behavior Change Methods

**Figure 2:**
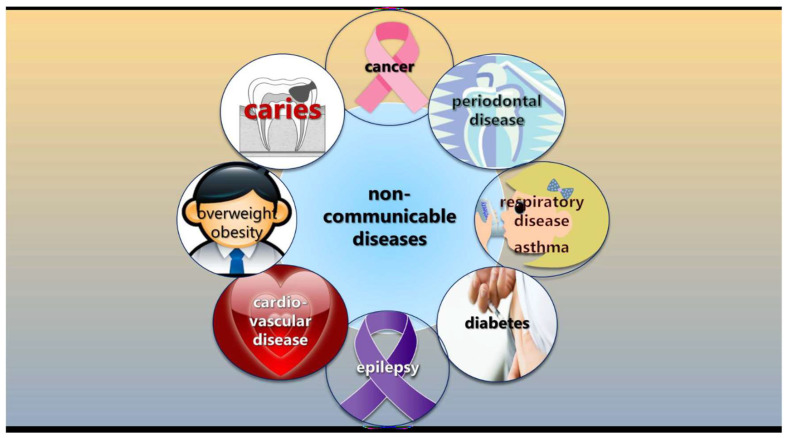
Caries is non-communicable disease, sharing risk factors with many other common conditions, such as unhealthy behaviors and diet.

The fact that caries is classified as a non-communicable disease (NCD) rather than a transmissible infectious disease gives dental professionals the opportunity to integrate their caries-preventive efforts with general health promotion according to the common risk factor approach [45,46]. A non-communicable disease is not spread through infection or through other people but is typically caused by unhealthy behaviors such as physical inactivity, harmful use of tobacco and alcohol, and unhealthy diets (Figure 2). This approach also calls for the adoption and incorporation of behavior change theories in the caries-preventive toolbox, in particular focused on sugar intake, regular tooth-brushing, and fluoride exposure. In this context, dentists seem to be remarkably absent when it to comes to reducing sugar intake, at least among adults [47]. There are recommended best-practice tools to evaluate sugar consumption, such as food frequency questionnaires, 24 h dietary recalls, and food diaries, but, according to a survey in the UK, general dentists do not frequently use such tools to collect diet information [48]. The main obstacles seem to be time constraints and insufficient remuneration for the time spent.

The most commonly embraced behavioral change model in dentistry for individual caries prevention in the clinical setting is motivational interviewing (MI) [49]. MI is a patient/parent-centered method for enhancing intrinsic motivation and strengthening commitment for change. The core elements are engaging, focusing, evoking, and guiding [50]. Patients are the best experts on themselves, and dental professionals can support them towards a wanted behavior in terms of oral hygiene, diet, and smoking in a “horizontal” conversation. In systematic reviews, low-certainty evidence shows that one-to-one interventions in the dental setting can change and improve dietary behavior [51,52]. Furthermore, MI interventions seem to be the most effective method for altering health-related behaviors and to reduce caries in schoolchildren and adolescents [51,52,53]. With a focus on the early ages, there is moderate-certainty evidence that MI is beneficial in reducing new carious lesions in children with early-childhood caries [54]. It has also been suggested that the benefits of such MI interventions may extend beyond caries prevention to other oral and systemic diseases [53]. Although MI has been used to promote healthy behaviors across a broad range of NCDs, its global use in everyday dentistry is unclear. In some countries, for example, Norway, education and policy support the use of MI, and all dental professionals receive training at varying depth, from lectures alone to lectures combined with courses, role-play, and supervised training in clinical practice. Yet only a minority of dentists seem to be confident in its use, which, of course, hampers the outcome [55]. Thus, an extended emphasis on the motivational interviewing technique in the dental curriculum has the potential to modify awareness and behavior for fluoride use and sugar snacking. It is, however, important to point out that MI is not a standalone measure in preventing caries in disadvantaged groups. A recent study conducted among high-caries-risk children and their families has shown that the combination of a behavior change technique and conventional caries prevention enhanced the outcome in comparison with MI alone [56]. This illustrates that a skill mix is needed for effective caries prevention.

## 2. Limitations and Comments

As this review is a narrative opinion, no formal and structured search of the literature was conducted, and the references were subjectively selected to support statements in the text. The starting point was the practicing everyday clinician, and this paper touches only on a few selected problems in caries prevention for children, with potential room for improvement. For example, this paper does not discuss the role of educational institutions and a universal core curriculum in cariology that could bridge the gap between preventive and restorative dentistry [57]. Other areas of interest not addressed here are the maintenance of a diverse and health-associated dental biofilm and AI-assisted caries detection and risk assessment. There are certainly also knowledge gaps in the literature, and we probably know less than we think we know [58]. Thus, further research in cariology is required, in particular on how to manage children with the highest caries risk and caries activity, although this type of research has legal and ethical constraints. An ideal scenario is that adequate research funding come from non-biased sponsors.

## 3. Conclusions

The prevention of caries in children is difficult and complex, but dentists do not fully utilize the tools that are available. Proper education and training in caries risk assessment, behavior change models targeted at oral hygiene and sugar, and collaboration with primary healthcare and local school authorities are avenues that aid caries prevention and can overcome the limited access to dental care in underserviced and remote communities. Further research is needed to unveil the benefits, health economics, and patient-perceived value of caries prevention in clinical practice. Preventive measures directed to children with the highest caries risk are still largely a knowledge gap, partly because this kind of research is associated with delicate practical and ethical considerations.
